# A Case of Transient Local Anesthetic Induced Bilateral Vocal Cord Palsy

**DOI:** 10.1155/2015/379258

**Published:** 2015-06-17

**Authors:** M. Rafiq, U. Al-Zoraigi, S. Alzahrani, Y. Alabdulkarim

**Affiliations:** ^1^Department of Anesthesia, King Fahad Medical City, Riyadh, Saudi Arabia; ^2^Department of Surgical Specialties, King Fahad Medical City, Riyadh, Saudi Arabia

## Abstract

We report a rare case of bilateral vocal cord palsy following total thyroidectomy with successful extubation within 12 hours. The patient is a 33-year-old lady who underwent uneventful total thyroidectomy for compressive symptoms. Thirty minutes after extubation, she developed stridor and the flexible laryngoscopy showed bilaterally adducted vocal cords. The patient, thus, was reintubated and after 12 hours she met the extubation parameters and so she was extubated successfully. The repeated flexible laryngoscopy showed normal vocal cords. A review of the literature revealed limited information on this transient condition.

## 1. Introduction

Thyroidectomy is the most common endocrine surgical procedure performed with its unique set of complications. Recurrent laryngeal nerve (RLN) palsy is the most unique and dreaded complication associated with the procedure having long term consequences. Permanent nerve palsy may occur in 0%–2.1%, with an average of approximately 0.5% to 1%. Temporary palsy varies from 2.9% to over 10% [1–3].

Mechanisms of injury to the nerve include complete or partial transection, traction, or handling of the nerve, contusion, crush, burn, clamping, misplaced ligature, and compromised blood supply [4, 5]. Local anesthetic induced RLN palsy is a rare condition but few cases have been reported in literature [6].

It is possible to block the recurrent laryngeal nerve with local anesthetic which is used as a treatment option in patients with adductor spasmodic dysphonia, which is a neurogenic condition characterized by continuous hyperadduction of vocal cords. RLNI is a major concern in thyroid and parathyroid surgery. Therefore, methods that can reduce the incidence of this complication are of great interest. We present a case of local anesthetic induced bilateral recurrent laryngeal nerve palsy.

## 2. Case Report

A 33-year-old female weighing 65 kg with a diagnosis of Hashimoto's thyroiditis 2 years ago was electively booked for total thyroidectomy under general anesthesia after she started to complain of mild compressive symptoms. Patient had no medical illnesses and euthyroid state was maintained with 50 *μ*g of levothyroxine. Computed tomography showed a thyroid gland of around 4 cm by 5 cm in size without any retrosternal extension. Flexible laryngoscopy prior to surgery was unremarkable. Preoperative anxiolysis was achieved with 2 mg of midazolam intravenously. Anesthesia was induced with 150 mg of propofol and 150 *μ*g of fentanyl and 40 mg of rocuronium was given intravenously to facilitate tracheal intubation. Airway was secured with size 7.0 mm I.D. endotracheal tube by direct laryngoscopy in the first attempt. Anesthesia was maintained on desflurane 4%–6%. Patient received paracetamol 1 gram, diclofenac sodium 75 mg, dexamethasone 8 mg, and granisetron 1 mg intravenously during the procedure. Surgery concluded uneventfully in 2 hours. Hemostasis was achieved and the recurrent laryngeal nerves were identified and spared. The strap muscles were approximated in the upper half to allow for drainage. After closure, the surgical wound was infiltrated in the subcutaneous tissue with 0.25% bupivacaine 5 mL and morphine 6 mg IV was given at the end of procedure. Patient was extubated while being awake at the end of the procedure with visualization of normally placed vocal cords. Patient was shifted to postanesthesia care unit (PACU) while being fully awake, breathing normally with stable vitals and normal vocalization. Patient remained stable for 30 minutes in the PACU when she started complaining of difficulty in breathing and vocalization which increased gradually. Inspiratory stridor was noticed and drop of Spo2 from 99% to 92% occurred which was managed by CPAP of 7-8 cm of H_2_O with 100% oxygen. Otolaryngologist was consulted and a diagnosis of bilateral adducted vocal cords secondary to RLN palsy was made by flexible laryngoscopy with absence of any airway or glottic edema. Calcium level at this point was 1.9 mmol/L (normal range 2.09–2.54 mmol/L). A decision was made to intubate and ventilate the patient and reassess the case after 12 hours in the ICU. Extubation trial after 12 hours was successful and flexible laryngoscopy showed normally placed vocal cords. Patient was breathing and vocalizing normally with normal nerve conduction tests. After excluding all causes of recurrent laryngeal nerve paralysis and the typical onset and recovery of the symptoms, a diagnosis of local anesthetic induced bilateral recurrent laryngeal nerve paralysis was made. Patient was discharged after a week in a stable condition with no complaints.

## 3. Discussion

The incidence of injuries to the recurrent laryngeal nerve has been reported between 1% and 2% from different thyroid surgery centers when performed by experienced neck surgeons. This incidence is higher when thyroidectomy is performed by a less experienced surgeon [1, 2] or when thyroidectomy is done for a malignant disease.

Mechanisms of injury to the nerve include complete or partial transection, traction, or handling of the nerve, contusion, crush, burn, clamping, misplaced ligature, and compromised blood supply [3, 4]. In unilateral RLNI, the voice becomes husky because the vocal cords do not approximate with one another. Dysphonia starting on the 2nd–5th postoperative days is commonly due to edema, whereas traction injury of the nerve and damage of axons may result in dysphonia lasting up to 6 months. Dysphonia continuing after 6 months is commonly permanent and is caused by cutting, ligating, or cauterization of the nerve [7]. Bilateral RLNI is much more serious because both vocal cords may assume a median or paramedian position and cause airway obstruction and tracheostomy may be required. Accidental transaction commonly occurs at the level of upper two tracheal rings, where the nerve closely approximates the thyroid lobe in the area of Berry's ligament [4, 5]. Although it is a very rare cause, local anesthetic induced bilateral recurrent laryngeal nerve palsy has been reported before in literature [6]. After removal of the thyroid gland, the nerve stands more exposed and near to the skin surface. The probability of local anesthetic coming into contact with nerve and blocking conduction increases.

Preintubation laryngoscopy and subsequent assessment have revealed that vocal fold injury can occur in approximately 30% of endotracheal intubations and 4% of postoperative RLNP is attributable to intubation injury. Accurate preoperative assessment and consideration of other causes of dysfunction including hyperaemia related to gastrooesophageal reflux, Reinke's oedema, and chronic laryngitis are crucial to reporting true surgical RLNP rates [8]. In our case, the flexible laryngoscopy after surgery showed absence of any edema, trauma, or hyperaemia in and around the vocal cords. The onset of the symptoms which started within one hour of the completion of surgery and injection of local anesthetic and complete resolution of symptoms within twelve hours, with confirmation by flexible laryngoscopy, again pointed towards a transient etiology.

It is thus concluded that local anesthetic injection at surgical site can be one of the possible causes of bilateral recurrent laryngeal nerve injury and should be considered as a probable differential diagnosis. The routine use of local anesthetic infiltration after thyroidectomy should be reviewed because of the confounding of the diagnosis of recurrent laryngeal nerve palsy and resultant morbidity.
